# Mentoring Students as a Professional Development Opportunity: Reflections of Early Career Dental Faculty

**DOI:** 10.1111/eje.70019

**Published:** 2025-07-15

**Authors:** Melanie J. Aley, Melinda L. Lawther, Patrick Westhoff, Ashleigh S. Ayo, Tabitha Acret, Jacqueline Biggar, William Carlson‐Jones, Kyle Cheng

**Affiliations:** ^1^ Sydney School of Dentistry, Faculty of Medicine and Health The University of Sydney Darlington Australia

**Keywords:** early career faculty, faculty development, mentoring

## Abstract

**Introduction:**

Mentoring oral health students as a faculty mentor potentially yields numerous benefits for the mentor's professional development, and very little is known about the role mentoring plays in the professional development of early career faculty. This program's aim was to explore the impact of mentoring oral health students on the professional development of seven early‐career dental faculty members.

**Materials and Methods:**

Seven early‐career faculty members were engaged as mentors to provide additional support to oral health students. Throughout the mentoring programme, each faculty mentor reflected on their experience and on completion met to discuss experiences. By exploring the reflective data, the common themes from the programme were identified.

**Results:**

The faculty were unfamiliar with providing academic mentoring for students and learned to develop important personal qualities such as openness and patience. Faculty identified a sense of ‘imposter syndrome’ in their role as new faculty and experienced conflicting feelings: guilt associated with time pressures, lack of perceived usefulness, and pride and satisfaction from the rewarding experience.

**Discussion:**

It appears that mentoring students can support faculty development, feasibly translating into improved teaching abilities and enhanced interpersonal competencies. Early‐career faculty identified further opportunities for faculty development, focused mostly on student wellbeing and study strategies.

**Conclusion:**

The mentoring program demonstrated that supporting early‐career oral health faculty helped facilitate their transition from clinical practice to academia. By providing a structured environment for growth and reflective practice, the program significantly contributed to the mentors' professional development.

## 1 Introduction

Educational literature has explored the journey many early career faculty face as they transition from professional practice into academia. For professional programmes, many early career faculty are recruited for their clinical experience in the professional practice of their discipline. The challenges they face, however, are aligning their practitioner expertise with evidence‐based teaching practices in higher educational environments [1, 2]. Hollywood and colleagues [3] also report that many early career faculty are faced with many initial challenges during their transition, ranging from limited academic support, large workloads and a high burden of stress and anxiety.

Educating the next generation of dental professionals is contingent on the presence of a strong, skilled dental faculty. However, dental education is encountering a shortage of faculty, posing a challenge for institutions [4]. There are substantial demographic and organisational changes taking place in Australian higher education institutions, including an aging workforce, increased casualisation of the workforce (i.e., teaching staff on casual contracts), increases in student enrolments, and changes to teaching and learning methods and delivery, all of which serve to impact on the appeal of academia as a career choice [5]. The conventional vertical pathway through undergraduate and graduate education, higher‐degree research and on into academia is eroding [6], whilst the demand for academics is growing [7]. This dilemma could be addressed through the career transition and employment of clinicians.

Skilled clinicians experience many challenges when transitioning into academia. The shift in identity can leave new academics feeling somewhat novel and vulnerable, which can impact on their confidence [8, 9]. Many early career faculty feel unprepared for the academic responsibilities they face, including teaching, research and institutional service, emphasising the need for professional development opportunities [10]. The transition from a practitioner to an educator role involves not only a shift in skill sets but also an adjustment in professional identity, which can be disorienting for new faculty [11]. The change in careers is highlighted as a mostly positive one with strong commitment to students; however, recommendations have been made to support new academics in their employment by having role models, realistic expectations as well as staff development initiatives in place for supporting the process [9, 10, 11, 12, 13]. Despite the differences in skills required to be a clinician and an academic, with this supportive environment in place, early career faculty can begin to thrive.

There is an obvious need for additional support in meeting the professional development requirements of early career faculty as they transition from practitioners to educators. Various formats have been reported from formal postgraduate education, informal learning and creating communities of practice. Bennett et al. [14] discussed the benefits of further education relating to teaching and leadership in supporting early career faculty in their professional development and work toward promotion. However, it has also been suggested that professional development that is integrated into everyday academic activities is also important [15, 16]. Through reflective practice, early‐career faculty can examine and analyse their teaching experiences and gain valuable insights for quality improvement [17]. Communities of practice (CoPs), defined as ‘groups of people who share a problem or interest in a particular topic, and help to build on individual's knowledge and expertise in an area’ are also effective programs to support early career faculty in their transition [18]. CoPs can provide an opportunity for peer conversations about personal reflections on teaching, for shared understanding [17]. Early career faculty that participated in CoPs were found to develop a greater awareness of Scholarship of Learning and Teaching (SoTL) [19].

Mentoring students is an important role for faculty. Mentoring students in higher education had numerous benefits, including improved student academic performance in the form of higher grade point averages, creation of an easier transition for the students into higher education, and the building of resilience and persistence in their studies [20]. Further positive outcomes identified included a greater sense of belonging, increased capacity for socially responsible leadership, deeper and more strategic learning approaches, and a greater level of self‐confidence in professional skills and abilities [20]. Dolan and Johnson [21] also found benefits for academic mentors, creating improved cognitive and socio‐emotional growth, communication and teaching skills.

The research gap in oral health faculty career development as mentors is evident in the limited literature addressing specific challenges and opportunities for new early career faculty. While mentorship is recognised as crucial for mentees in the academic setting [22] and within dental schools [11, 23] there is limited research regarding oral health faculty as mentors [24]. Nursing, pharmacy and biomedical science have explored the benefits of mentoring on both the mentee and mentor in higher education, but there was no further research available in the dental education setting [24, 25, 26, 27, 28]. When looking at oral health mentorship, research literature can be found on the benefits of mentoring within the dental setting of graduated mentees [29] however, there is a gap in the research for the unique context of oral health faculty and its impacts on their career development.

Further exploration is warranted to understand the distinctive needs of faculty mentors in the dental education setting and the outcomes for career development in early career faculty. Closing the gap can help us understand the dynamics of mentoring in an oral health academic setting, ultimately contributing to the optimisation of mentorship and career development in the oral care setting for early career oral health faculty. This research paper aims to investigate and analyse the impact of mentoring oral health students on the early career faculty development of faculty mentors. Through reflective analysis, the study aims to identify the specific ways in which mentorship influences professional skill acquisition and overall career development, contributing valuable insights to educational practices of early career faculty.

## 2 Methods

### 2.1 Context: The Mentoring Program

The mentoring program was introduced within the Faculty of Dentistry at an Australian university, specifically targeting 19 Bachelor of Oral Health (BOH) students identified as requiring additional support. This classification included students who had faced challenges such as failing units of study, not meeting hurdle requirements, or being identified as requiring additional support during their clinical placements by their educators. Students were identified from all year levels (1–3) and were offered the opportunity to voluntarily participate in the mentoring program. Of the identified students, 15 demonstrated a willingness to engage in mentoring and accepted the offer. Subsequently, these 15 students were assigned to seven early‐career education‐focused faculty members who volunteered to participate as faculty mentors in the program. There was no matching process for mentees and mentors, given all mentors were faculty and had the capabilities to provide the support required.

The mentoring program was designed using a traditional, formal approach [30] providing a platform for both faculty mentors and student mentees to achieve the goals of enhancing their skills and abilities, sharing experiences and gaining insights into student‐related issues. An introductory information session was conducted for student mentees, outlining the program's objectives, expectations and the benefits of mentoring. Alongside this, faculty mentors underwent an online training program to familiarise themselves with expectations and receive guidance on suggested activities. During the training, mentoring was defined as a mutually beneficial relationship in which an experienced person assists another in developing specific skills and knowledge that will enhance the less‐experienced person's professional and personal growth. Additionally, a comprehensive resource guide was developed for both student mentees and faculty mentors.

The mentoring program ran for a 13‐week semester. During the initial mentoring session, mentees discuss their challenges, and, with guidance from their mentors, create a personalised learning plan. Meeting logistics, such as location, frequency (once a fortnight was suggested) and duration, were also established, noting the program was designed to be flexible around the student mentee's commitments. Subsequent mentoring sessions focus on mentors providing ongoing support, acting as sounding boards, sharing knowledge, offering encouragement, guidance and constructive feedback. The faculty mentor periodically reviews the progress of the learning plan, provides additional strategies and ensures the mentee reflects on and evaluates their progress every fortnight. Student mentees are encouraged to raise any issues, such as study habits, assessments, or clinical education feedback, during these sessions. Anticipating upcoming weeks and strategising for success are also integral aspects of the ongoing mentoring discussions. In the final mentoring session of the semester, faculty mentors confirm the achievement of mentees' goals and collectively reflect on the progress made throughout the semester. Student mentees are prompted to consider how they will continue their learning in the subsequent semester. At the conclusion of the semester, faculty mentors participate in a debriefing session where the BOH Program Director assesses the program's success and identifies areas for improvement. This evaluation process ensures the continuous enhancement of the mentoring program to better serve the needs of BOH students.

### 2.2 Data Collection

Faculty mentors completed a reflection at the beginning, midpoint, and end of the 13‐week mentoring programme, using an online form. The Gibbs [31] reflective cycle was used to structure the prompts for reflecting on their experience as mentors. All faculty mentors were familiar with reflective practice, given their prior experience as dental clinicians. Faculty mentors were prompted to reflect on their mentoring meetings and respond to six questions: What happened? What have you been feeling and thinking about during the meetings? What was good and bad about the experience? What sense can you make of the experience? What have you learned? What will you do? Faculty mentors met face‐to‐face at the completion of the mentoring programme to debrief and discuss their experiences, what impact the mentoring had on them, further professional development requirements and recommendations for improving the programme.

### 2.3 Data Analysis

An inductive thematic analysis approach [32] was used for the qualitative analysis of this data. The focus group recording was listened to, and the transcription analysed for errors, allowing for an initial familiarisation with the data. The initial codes were identified from the focus group and written reflections using NVivo14, used with permission from Lumivero [33]. The codes were then grouped together, checked for emerging patterns, and the initial themes were identified. The ongoing refinement of the themes took place during discussion among three of the early career faculty with a critical reflective lens. Finally, the three overarching themes were checked with reference to relevant literature during the collaborative writing process.

### 2.4 Ethical Considerations

As this study is based on reflective practice, with all data drawn from the authors' own lived experience and professional development, no ethics approval was required. No other individuals were studied or represented without consent, and ethical standards were maintained through reflexivity, voluntary participation and anonymisation of individual responses.

## 3 Results

### 3.1 Participants: Early Career Faculty Mentors

The participants exhibited diverse levels of mentoring experience. One participant demonstrated expertise in mentoring new graduate clinicians, while another had experience mentoring new clinical staff during their orientation process and assisting staff facing concerns. Additionally, one participant had prior experience mentoring dental students, two participants engaged in informal mentoring with colleagues and students, and two participants had no previous mentoring experience.

All participants held qualifications as oral health practitioners, with three possessing post‐graduate qualifications in education. Despite their qualifications, all participants were early‐career faculty, with their tenure in academia ranging from 1 month to 2 years. Outside of university teaching, one participant had experience teaching in continuing professional development courses, and another had over 2 years of experience as a yoga teacher. Concerning prior mentoring training, only two participants had received formal training to become mentors. When asked to rate how prepared they felt prior to the commencement of mentoring sessions on a 5‐point Likert scale (1 being ‘not prepared at all’ and 5 being ‘very well‐prepared’) for the mentoring sessions, one participant gave a score of 1, two participants gave a score of 2, and four participants rated themselves a 3.

### 3.2 Unfamiliar Role

There were varying levels of experience with mentoring. One early career faculty member had experience with mentoring clinically, two briefly mentored USYD dentistry students, and the other faculty member had no mentoring experience. Four out of the seven early career faculty members expressed concerns, feeling unfamiliar with the role of mentoring.

I have little experience with one‐on‐one mentoring, so felt a little perturbed.

I have not been in a mentoring situation like this. I felt a little overwhelmed and unsure of what to say or do for the student. I felt like what I was telling her might not be correct and I would second guess myself a lot.

These concerns were expressed at beginning of the program, after the initial meeting with the mentee. The unfamiliar role of mentoring seemed to be coupled with wavering confidence in providing a good mentoring experience to the students, ability to support the students in providing adequate support and feeling not prepared for the meetings.

I don't feel confident as a mentor and it's just I haven't done it so much.

I wish I prepared more for each session to kind of ask some questions that reflected on her problem.

One early career faculty felt the need to assist the mentee to achieve the goals set in one meeting:

[I] did feel a little unsure of myself at times and maybe this may have been in my preparation or my expectations of the mentoring program. I was able to give good examples of my clinical experience to the student. I think that I wanted to achieve everything I wanted in one meeting.

Reflection at the end of the program has brought attention to the understanding patience is needed throughout the mentoring process.

Openness was another quality that the early career faculty felt was important throughout the mentoring programme. Not every mentee had the same issues, and it was important to listen to their needs for the mentoring programme, whether it be working through academic‐focused challenges, or struggling in their lives outside of the BOH programme.

I have learned to be understanding and empathetic to individuality.

I had assumed the students may have wanted some advice around content and assessments, but each meeting focused more on how to establish good study habits and their want to be able to just talk through their feelings with someone in the industry.

Just kind of reminds me that, like everyone has their own things … everybody has their like battles. … So, you've just got to really keep that in front of mind.

Even though these important mentor qualities were realised early in the program, the unfamiliar mentoring environment and the lack of mentor confidence continued through the program and the feeling of ‘imposter syndrome’ developed.

### 3.3 Imposter Syndrome

It was discovered that upon reflection midway through the program, all early career faculty experienced a feeling of ‘imposter syndrome’ or not being ‘good enough’ to positively impact students' lives, despite being qualified and accomplished. It was discussed that the early career faculty already had a feeling of self‐doubt as they are in the early stages of developing their own understanding and expertise in teaching, being at the start of their professional academic journeys. From this self‐doubt, a lack of perceived usefulness was discussed.

I feel a little bit of imposter syndrome to be honest. I feel like I either don't have the skillset or am not “qualified” to be helping the student. I do my best in helping the student with giving my clinical experience and she seems to be taking it on board.

Despite the feelings of ‘imposter syndrome’, there were faculty mentors who reported a gain in confidence after having multiple sessions with their mentees.

I feel as though the more meetings we have, the more confident I am discussing issues and concerns with my mentee.

Many faculty mentors felt they had started to build a positive professional relationship with their student mentee.

I feel as though our communication has improved as my mentee feels more confident and comfortable speaking with me about their issues or concerns, or just life in general.

There was also discussion of faculty mentors beginning to have more understanding and compassion that everyone's learning journey can be quite different.

### 3.4 Personal Development

As the program approached its end, faculty mentors conducted follow‐up meetings, varying from 1 to 8 sessions after the initial meeting. Despite experiencing imposter syndrome midway through the program, many faculty mentors continued to grapple with this feeling. Toward the program's conclusion, fresh challenges emerged in the form of guilt due to time constraints and the challenge of how the meetings were arranged. As the semester was starting to become busier, both faculty mentors and student mentees found themselves increasingly occupied, making it challenging to schedule meetups. Students faced heightened demands with additional assessments and clinical placements for most of their week. Similarly, early career faculty found themselves occupied with preparation of lectures and other teaching commitments, along with four out of the seven faculty being part‐time employees, so finding mutual times to meet up was difficult.

I had some guilt in the last few weeks because I wasn't able to provide the time to spare with them.

Both myself and my mentee got progressively busier after week 9 and onwards due to exams, clinics, all day teaching etc.

The way in which meetings were arranged continued to be a struggle for most faculty mentors having meetings held either online or in‐person. Most faculty mentors found that using an online platform to hold meetings became a struggle as student mentees would prefer to turn their camera off, which discouraged their participation in the meeting. Holding meetings online like this reduced the building of rapport between faculty mentor and student mentee and some mentors found that they would fill silence through holding the conversation single handedly.

I think I should of made them face to face, zoom didn't really work. Moving forward I will do face to face next time as I feel it helps foster a better relationship.

Nevertheless, despite facing challenges throughout the entire program, faculty mentors discovered the overall experience to be fulfilling. Seeing student mentees gain confidence as they came to the end of the program was rewarding and built a sense of pride and satisfaction among the faculty mentors.

I have learned more about my mentee and understand them better, now we have an established relationship. I have learned to be more empathetic to understand people's struggles, particularly with study and learning. Not everyone learns/understands the same way and thats ok.

I have come to realise that these students that I supported never really saw themselves as someone who was bottom of the class or that poor student that needs help. Both my mentees saw this experience as a way to really help them through the semester and not as that poor student.

Faculty mentors derived a sense of purpose from recognising selflessness and gaining an understanding of an individual's personal struggles. Anecdotally, every student mentee had individual motivations for enrolling in the program, along with their unique personal challenges and with this, faculty mentors experienced fulfilment in delivering this mentoring program to the students and witnessing their academic development.

## 4 Discussion

This study explored how mentoring students contributed to the personal and professional development of early career dental faculty, for what appears to be the first time. Being involved as a faculty mentor for oral health students was designed to support professional development as it provided an opportunity to work one‐on‐one with students, developing skills in problem solving, communication and working with others. Trained mentors have reported better communication skills and professional development [34] and establishing connections, affirmation and career development [35]. There is very little reported in the literature about the benefits of mentoring for faculty mentors, with developing relationships, rewarding and giving back noted in a study of occupational therapy mentoring programmes [36].

Reflection of the early career faculty plays a significant role during the mentoring process. By building in the Gibbs Reflective Cycle [31] at multiple stages, the mentoring programme offered the early career faculty high‐information feedback, which Pieper et al. [37] showed to enhance the quality of reflection for the faculty. Brookfield [17] also highlights this, stating reflective practice allows the early career faculty to explore their own teaching experiences, thus improving the quality of their teaching. They discovered unfamiliarity with their academic position, realisation of imposter syndrome, increased awareness of their own teaching practices, understanding of students' personal challenges, and ultimately their own personal development.

The unfamiliarity of their academic position, and as a faculty mentor, was a challenge for the early career faculty. Alleviating this unfamiliarity is a challenge for higher education institutions and their leaders. There is an assumption that being an experienced clinician prepares new faculty for their academic roles [8]; however, research has highlighted that clinicians do not necessarily understand what academic careers entail [38]. Similar professionals such as nursing have identified the need for a better induction and orientation programme for novice faculty [8]. Our faculty completed a university‐wide orientation programme, including a series of online and in‐person training modules and an onboarding process within the local team. Staff were intentionally supported through the University's academic planning and development cycle by a supervisor and assisted with setting goals and a development plan. Wendler and colleagues [39] have identified that early career faculty need to be ‘intentionally supported’ through formal orientation, socialisation, mentoring and resources to be successful. Strategies used by early career faculty transitioning from clinical practice include doing further study such as postgraduate certificates in education [8]; this was a strategy that was used by some of the faculty; however, often not through choice but requirements of their probationary period as new education‐focused faculty.

Imposter syndrome was experienced by every early career faculty member in the program. This is reported by Hutching [40] revealing this phenomenon in the wider higher education faculty. The early career faculty in the mentoring program reported these feelings, leading to a lack of confidence and self‐doubt in their abilities, similarly to those found by early‐career physicians [41]. To combat imposter syndrome, early career faculty have had to cultivate self‐belief. This self‐confidence has empowered them to trust their expertise and judgement, enhancing their overall academic presence and leadership capabilities. Feelings of uncertainty and a lack of self‐confidence often hinder the transition for early career faculty into academia, which can impede the adoption of an academic identity and limit career advancement opportunities [8, 42]. Self‐confidence is a critical component of identity construction [43] and is seen as essential for successful transition and career progression [44]. For early career faculty, self‐confidence is a vital personal quality. It enables them to navigate the challenges of new roles, engage fully with their academic identity, and seize opportunities for advancement. Building self‐confidence helps to overcome initial uncertainties and fears, paving the way for a successful and fulfilling academic career. These qualities have been identified and cultivated through this mentoring program.

As evidenced by this study's results, the mentoring program has facilitated the development of key personal qualities in early career faculty. The challenge of assuming a mentorship role without prior experience has fostered the quality of openness and empathy. This openness is evident in their heightened receptiveness to new ideas and diverse perspectives, fostering empathy, which is essential for both their professional and personal growth. In higher education, an empathetic view of the student's experience is essential for addressing retention issues [45]. This process involves understanding the individual student's experience and the institutional structures that may hinder success, and reframing student success around their potential. Supporting student mentees within the mentoring program requires a creative, optimistic and empathetic approach that considers both individual attributes and organisational structures while recognising and overcoming biases. This mindset fosters innovative and unbiased insights essential for effective mentoring. Empathy, identified as a key personal quality, further aids in the development of the faculty's identity as a student‐centred educator [46]. This approach to university teaching is embedded in the current philosophy. Meyers et al. reinforce empathy as an important teacher quality to identify and remove student obstacles to learning. Through providing mentoring to the students, the early career faculty can support the students in a way that can increase the chances of success in the program together with improving the quality of their teaching [46].

Reflective practice also plays a role in developing the faculty's empathy and student‐led teaching practices. Kligyte [47] showed that reflection was an important tool for the academic to take a more student‐centred approach to teaching. The early career faculty in this study found the mentoring experience to be valuable in informing their teaching practices when they gained unique insight into the understanding of students' individual personal challenges. Through this, the faculty are able to alter their teaching strategies to enhance the students' experience [47]. Further to this, reflection prompted early career faculty to balance their own responsibilities with those of mentoring students, which has underscored the importance of effective time management. Early career faculty must juggle the demands of marking assessments, developing teaching materials and maintaining a healthy work‐life balance. Mastery of time management has become crucial, enabling them to efficiently manage their workloads and mentor commitments, thereby reducing stress and enhancing productivity. Time management is an important personal quality that helps early career faculty thrive in their multifaceted roles [48] and has been highlighted upon reflection on the mentoring programme.

The mentoring program has emphasised to the early career faculty the importance of their own professional growth. The development of their academic identity is imperative for building confidence and ultimately fostering career progression. This program has identified the importance of the current induction programs in place to support early career faculty through their journey as an academic and the need to establish their own academic community [48] in support of their ongoing support and development. While this mentoring program has shown to be able to provide support to the early career faculty, it is proposed that this could be enhanced in a few ways. By building a CoP in the mentoring program, it facilitates opportunities to engage in conversations reflecting on teaching and challenges [17]. Early career faculty also suggested access to a pool of resources to offer students and an opportunity for further training courses such as formal mentoring training and Mental Health First Aid training. We plan to continue offering the mentoring program, with plans to offer it to any interested students. Given the potential workload implications of such an endeavour, we are also considering trialling a group mentoring structure.

### 4.1 Strengths and Limitations

The research provides insight into early career faculty abilities around mentorship and identified areas for improvement where professional development can be undertaken. The study was cost effective and reliable, and the findings can be used to improve such programmes and young academics' career satisfaction.

The major limitation of this study is the cohort size of participants (seven) and that all participants were working at the same University with access to the same resources. The results therefore may not be generalisable to all early career dental faculty. Albeit a small sample, the reflections provide valuable insights into an underexplored area of faculty development, and this new evidence can be a steppingstone for further research in this space.

## 5 Conclusion

The mentoring program demonstrated that supporting early‐career oral health faculty helped facilitate their transition from clinical practice to academia. By providing a structured environment for growth and reflective practice, the program contributed meaningfully to the faculty mentors' professional development. The faculty mentors' experiences underscored the importance of patience, empathy and individualised attention in fostering effective faculty mentor–student mentee relationships.

Despite initial challenges and feelings of imposter syndrome, the faculty mentors' journey through the program revealed a transformation in their confidence and capabilities. Through reflective practices and regular meetings, faculty mentors developed crucial qualities such as openness, patience and empathy. These qualities enhanced their mentoring skills, teaching practices and interpersonal competencies. The reflective process allowed them to navigate their emotions, identify areas for self‐improvement and develop essential qualities that enhanced their teaching and mentoring skills. Additionally, the program fostered a sense of community and collaboration among early‐career faculty members, promoting a supportive academic environment. The validity of our conclusions is supported by a rigorous reflective approach, which included structured, collaborative reflection and critical dialogue among authors. These processes enhanced the trustworthiness of our insights and ensured that the themes presented are grounded in authentic shared experience, with potential resonance for similar educational contexts.

The program's success highlights the need for similar initiatives in dental education to bridge the gap between clinical practice and faculty professional development. By addressing the distinctive needs of early‐career oral health faculty and providing them with the necessary support and resources, institutions can ensure the ongoing advancement of the dental profession. Future research should continue to explore the long‐term impact of mentoring on faculty mentors, ultimately contributing to the optimisation of mentorship and career development in the oral health faculty setting.

## Conflicts of Interest

The authors declare no conflicts of interest.

## Data Availability

Research data not shared.
